# Real world impact of added FFR-CT to coronary CT angiography on clinical decision-making and patient prognosis – IMPACT FFR study

**DOI:** 10.1007/s00330-023-09517-z

**Published:** 2023-03-15

**Authors:** Leonie M. Becker, Joyce Peper, Bram J. L. A. Verhappen, Laurens A. Swart, Admir Dedic, Willem G. van Dockum, Martin van der Ent, Kees-Jan Royaards, André Niezen, Jan-Hein J. Hensen, Jan-Peter van Kuijk, Firdaus A. A. Mohamed Hoesein, Tim Leiner, Tobias A. Bruning, Martin J. Swaans

**Affiliations:** 1 Department of Cardiology, St. Antonius Hospital, Koekoekslaan 1, 3435 CM Nieuwegein, The Netherlands; 2 Department of Radiology, University Medical Center Utrecht, Utrecht, The Netherlands; 3 Department of Cardiology, Erasmus Medical Center, Rotterdam, The Netherlands; 4 Department of Cardiology, Noordwest Ziekenhuisgroep, Alkmaar, The Netherlands; 5 Department of Cardiology, Maasstad Hospital, Rotterdam, The Netherlands; 6 Department of Radiology, Maasstad Hospital, Rotterdam, The Netherlands; 7 Department of Radiology, Mayo Clinic Rochester Minnesota, Rochester, USA

**Keywords:** Stable angina, Coronary artery disease, Computed tomography angiography, Fractional flow reserve, myocardial, Coronary angiography

## Abstract

**Objectives:**

The addition of CT-derived fractional flow reserve (FFR-CT) increases the diagnostic accuracy of coronary CT angiography (CCTA). We assessed the impact of FFR-CT in routine clinical practice on clinical decision-making and patient prognosis in patients suspected of stable coronary artery disease (CAD).

**Methods:**

This retrospective, single-center study compared a cohort that received CCTA with FFR-CT to a historical cohort that received CCTA before FFR-CT was available. We assessed the clinical management decisions after FFR-CT and CCTA and the rate of major adverse cardiac events (MACEs) during the 1-year follow-up using chi-square tests for independence. Kaplan–Meier curves were used to visualize the occurrence of safety outcomes over time.

**Results:**

A total of 360 patients at low to intermediate risk of CAD were included, 224 in the CCTA only group, and 136 in the FFR-CT group. During follow-up, 13 MACE occurred in 12 patients, 9 (4.0%) in the CCTA group, and three (2.2%) in the FFR-CT group. Clinical management decisions differed significantly between both groups. After CCTA, 60 patients (26.5%) received optimal medical therapy (OMT) only, 115 (51.3%) invasive coronary angiography (ICA), and 49 (21.9%) single positron emission CT (SPECT). After FFR-CT, 106 patients (77.9%) received OMT only, 27 (19.9%) ICA, and three (2.2%) SPECT (*p* < 0.001 for all three options). The revascularization rate after ICA was similar between groups (*p* = 0.15). However, patients in the CCTA group more often underwent revascularization (*p* = 0.007).

**Conclusion:**

Addition of FFR-CT to CCTA led to a reduction in (invasive) diagnostic testing and less revascularizations without observed difference in outcomes after 1 year.

**Key Points:**

• *Previous studies have shown that computed tomography–derived fractional flow reserve improves the accuracy of coronary computed tomography angiography without changes in acquisition protocols.*

• *This study shows that use of computed tomography-derived fractional flow reserve as gatekeeper to invasive coronary angiography in patients suspected of stable coronary artery disease leads to less invasive testing and revascularization without observed difference in outcomes after 1 year.*

• *This could lead to a significant reduction in costs, complications and (retrospectively unnecessary) usage of diagnostic testing capacity, and a significant increase in patient satisfaction.*

**Supplementary Information:**

The online version contains supplementary material available at 10.1007/s00330-023-09517-z.

## Introduction

Coronary computed tomographic angiography (CCTA) is a non-invasive test used to assess the presence and severity of coronary artery disease (CAD) [1]. CCTA has shown high sensitivity and negative predictive value, reliably identifying or excluding anatomically significant obstructive CAD [2]. However, for intermediate stenoses (50–90% diameter reduction), the relationship between diameter reduction and lesion-specific cardiac ischemia is not straightforward [1]. Invasive coronary angiography (ICA) with fractional flow reserve (FFR), the ratio of the blood pressure distal to proximal across a stenosis under hyperemic conditions, is generally used to assess the functional severity of individual atherosclerotic lesions [3–5]. When assessed with invasive measurements, the majority of intermediate stenoses detected by CCTA are not functionally significant, i.e., do not cause cardiac ischemia [1]. ICA and FFR are considered low-risk procedures, but are frequently performed and complications do occur [6].

Various studies have demonstrated that the diagnostic performance of CCTA, especially the specificity, can be significantly improved by computed tomography-derived FFR (FFR-CT), adding functional information to anatomical information derived from CCTA [5, 7–11]. FFR-CT is a non-invasive method which uses computational fluid dynamics principles to simulate invasive FFR [5]. The aim of adding FFR-CT to CCTA is to decrease the number of diagnostic ICA and FFR procedures in patients without obstructive CAD [12, 13]. Studies have confirmed that FFR-CT could lead to significant changes in clinical management, such as a reduction in ICA procedures [7–11, 14, 15]. Because FFR-CT can be applied to CCTA images without changes in imaging protocols, radiation dose, or medication, this improvement of diagnostic performance comes without additional burden or risks for the patient [5, 12].

Currently, limited data is available regarding the impact of FFR-CT on routine clinical practice. In clinical studies, patient selection and management generally adhere to strict protocols, and therefore do not represent routine practice. Clinical management comparisons for CCTA and FFR-CT are often conducted by first presenting CCTA images and then additional FFR-CT results to a specialist team, and comparing hypothetical patient management [7, 8]. None of this can be translated directly to real-world patient management. In this study, we assess the impact of the addition of HeartFlow FFR-CT to CCTA on clinical decision-making and patient prognosis in a routine clinical patient population.

## Methods

### Design and study population

This retrospective single-center cohort study included patients suspected of stable CAD for which they underwent CCTA as first coronary imaging in the Maasstad Hospital, Rotterdam, the Netherlands. All patients who received CCTA with FFR-CT between October 2018, when FFR-CT first became available in this hospital, and December 2020 were assessed for eligibility. As control group, we included a historical cohort of consecutive patients who underwent CCTA between January 2015 and September 2018, before FFR-CT was available.

Patients were included if they had at least one intermediate stenosis (diameter reduction ≥ 50%) on CCTA [16]. Patients were excluded if they were asymptomatic, were suspected of unstable angina, or had a history of coronary imaging (i.e., CCTA, ICA) or revascularization (PCI, CABG), one or more total occlusions on CCTA, non-interpretable CCTA, or a cardiac rhythm other than sinus rhythm during CCTA. Additionally, patients in the CT-FFR group were excluded if CT-FFR results had not been available for clinical decision-making. Approval of the local institutional human ethics review board was obtained. Data collection was anonymous and patients were not contacted. Therefore, the need for informed consent was waived (Medical research Ethics Committees United, registration number W21.076).

### Diagnostic tests

#### Coronary CT angiography

CCTA was performed using a Siemens Somatom Flash dual source 2 × 128 scanner between 2015 and August 2020, and a Siemens Somatom Drive dual source 2 × 128 scanner after August 2020. The CCTA acquisition protocol included a prospective electrocardiogram gated study with dose modulation in accordance with the Society of Cardiovascular Computed Tomography Guidelines on performance of CCTA and the local hospital protocol [17]. In brief, oral and/or intravenous beta-blockers were administered to obtain a heart rate ≤ 60 beats per minute. Immediately before image acquisition, 0.2 mg sublingual nitroglycerin was administered. CCTA reconstruction was performed using a dedicated post-processing workstation (syngo.via). The radiation dose in mSv was directly obtained from the CCTA report.

#### HeartFlow FFR-CT

CCTA images were transmitted to the HeartFlow core laboratory for FFR-CT-analysis. A quantitative 3-dimensional anatomic model of the aortic root and epicardial coronary arteries was generated and blood flow and blood pressure were computed under simulated hyperemic conditions. The results of FFR-CT measurements were provided throughout the 3-dimensional model of the coronary artery tree. The cutoff value of FFR-CT for hemodynamically significant stenosis was ≤ 0.80.

### Outcomes

As follow-up, all patient records were assessed until 12 months after CCTA. The safety outcome was major adverse cardiac events (MACEs), composed of all-cause mortality, aborted sudden cardiac death, myocardial infarction (MI), and unplanned (urgent) revascularization, within these 12 months.

Clinical management decisions were made according to standard hospital practice, following European Society of Cardiology guidelines [1]. Coronary segments were categorized according to the 16-segment model of the American Heart Association [18]. CCTA images were assessed by an imaging cardiologist and a radiologist. Clinical management decisions following CCTA were made by the treating cardiologist, although the CCTA report included recommendations. However, the decision to perform FFR-CT was made by the imaging cardiologist and radiologist assessing the CCTA images. Clinical management following FFR-CT results was discussed in a multidisciplinary team—the imaging team, which included an imaging cardiologist, an interventional cardiologist and a radiologist. Clinical management decisions regarding ICA-results and obtaining invasive pressure measurements were made by the interventional cardiologist. The interventional cardiologist could refer the patient to the heart team for multidisciplinary consultation. The heart team consisted of cardiologists and cardiothoracic surgeons from various hospitals across the region.

All data was extracted from medical records, which included the initial visit to the emergency department or outpatient clinic, diagnostic work-up, clinical management, occurrence of MACE, and follow-up appointments. If clinical management was missing or the patient could not be reached to discuss results or clinical management, the patient was excluded. Medical records were assessed by two independent researchers. Discrepancies were discussed and solved by mutual agreement.

### Statistical analysis

Categorical variables were expressed as numbers and percentages, while continuous variables were presented as mean and standard deviation in the case of normal distribution. Skewed continuous variables were presented as median and interquartile range. The outcomes between groups were compared using the chi-square test for independence. Kaplan–Meier curves were used to visualize the occurrence of safety outcomes over time. Patients with incomplete follow-up were censored at the last documented contact. *p* values of < 0.05 were considered significant. All statistical analyses were performed using R statistical software (www.r-project.org, version 3.4.2).

## Results

**Fig. 1:**
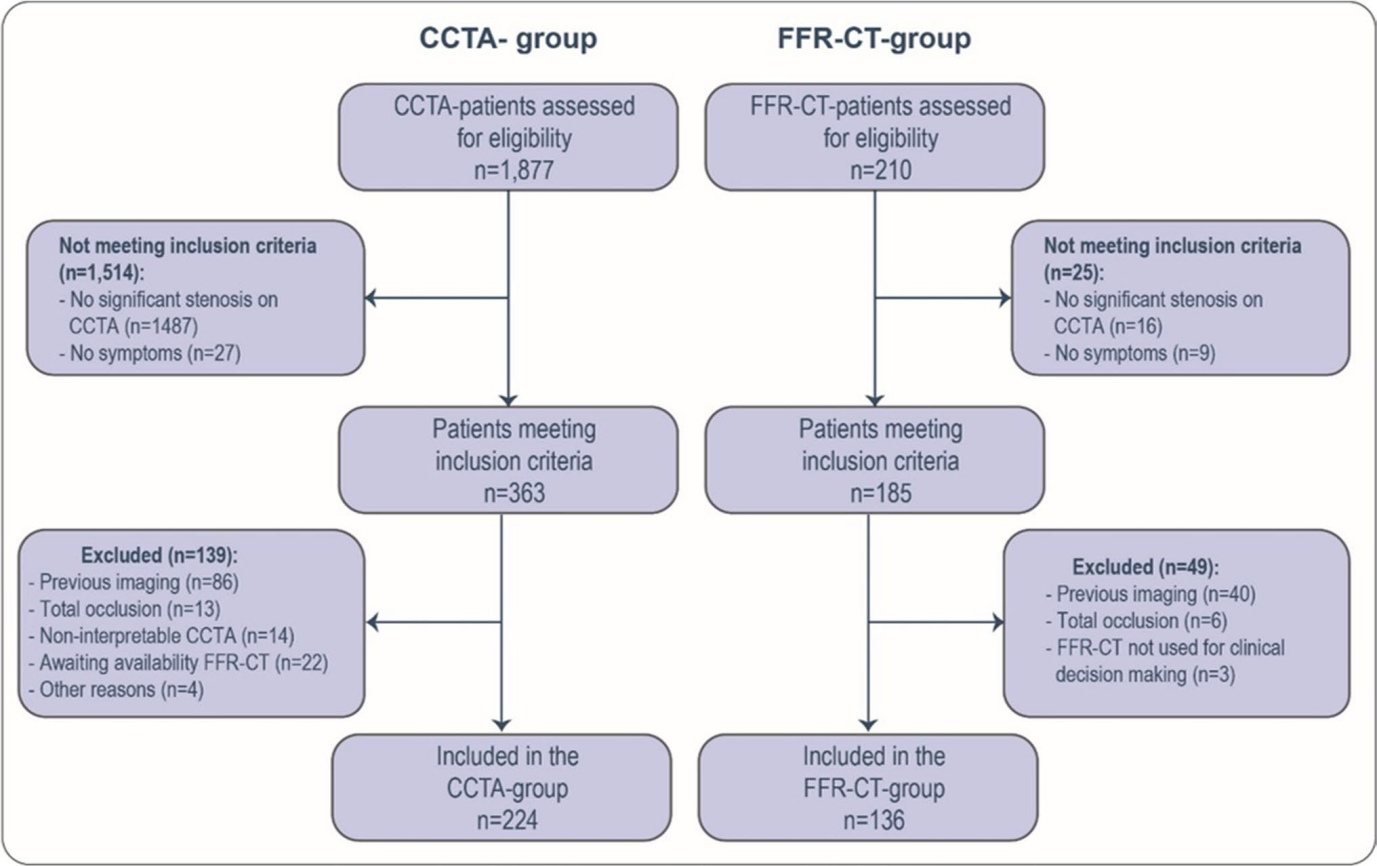
Inclusion flow chart. Flow chart of the selection of cases for analysis. Values are presented as *n*. CCTA, coronary computed tomography angiography; FFR-CT, computed tomography derived fractional flow reserve

In total, 360 patients with low to intermediate risk of CAD were included, 224 in the CCTA group and 136 in the FFR-CT group (Fig. 1). The mean age was lower in the CCTA group (58.4 ± 8.3 years and 60.9 ± 9.1 years respectively, *p* = 0.007). No other baseline differences were observed (Table 1). CCTA-quality was comparable in both groups.

**Table 1:** Patient- and coronary CTA characteristics of the study participants according to diagnostic strategy. Values are presented as *n* (%), or mean (standard deviation). *BMI* body mass index (weight in kilograms divided by the square of the height in meters), *CCTA* coronary computed tomographic angiography, *ECG* electrocardiogram, *FFR-CT* computed tomography–derived fractional flow reserve, *ICA* invasive coronary angiography, *SD* standard deviation, *LBTB* left bundle branch block, *EF* ejection fraction, *kVp* peak kilovoltage, *mSV* millisievert

Patient characteristics	CCTA (*n* = 224)	FFR-CT (*n* = 136)	*p*-value
Gender (male)	118 (52.7)	72 (52.9)	1.000
Age	58.41 (8.33)	60.93 (9.07)	0.007
BMI	28.96 (5.09)	28.09 (4.50)	0.101
Hypertension^a^	144 (64.3)	80 (58.8)	0.355
Hypercholesterolemia^b^	113 (50.4)	68 (50.0)	1.000
Diabetes mellitus	40 (17.9)	24 (17.6)	1.000
Current smoking	121 (54.0)	63 (46.3)	0.191
Diamond-Forrester score			0.679
Non-cardiac	122 (54.5)	69 (50.7)	
Atypical	77 (34.4)	53 (39.0)	
Typical	25 (11.2)	14 (10.3)	
Dyspnea on excertion	63 (28.1)	44 (32.4)	0.464
Rest ECG			0.456
Normal	159 (71.0)	87 (64.0)	
Aspecific ST/T deviations	31 (13.8)	26 (19.1)	
T-inversions	18 (8.0)	9 (6.6)	
ST-deviations	4 (1.8)	6 (4.4)	
LBTB	10 (4.5)	6 (4.4)	
Missing	2 (0.9)	2 (1.5)	
Left ventricle function (EF)			0.814
Normal (> 50%)	119 (53.1)	67 (49.3)	
Mildly-moderately abnormal (30–50%)	2 (0.9)	1 (0.7)	
Severely abnormal (< 30%)	0 (0.0)	0 (0.0)	
regional wall motion abnormalities	5 (2.2)	2 (1.5)	
Missing	98 (43.8)	66 (48.5)	
CCTA characteristics			
Scanprotocol–prospective (ECG triggered)	202 (90.2)	135 (99.3)	0.001
Scanprotocol–flash	28 (12.5)	6 (4.4)	0.018
CCTA quality			0.070
Good	183 (81.7)	123 (90.4)	
Moderate	40 (17.9)	13 (9.6)	
Poor	1 (0.4)	0 (0.0)	
Tube voltage (kVp)	121.70 (13.75)	117.62 (18.12)	0.017
Radiation dose (mSV)	6.65 (3.75)	5.08 (3.88)	0.025
Heart rate during CCTA	62.21 (9.62)	58.44 (6.42)	0.012

^a^Systolic blood pressure > 140 mmHg or diastolic blood pressure > 90 mmHg or the use of antihypertensive drug(s)

^b^Total cholesterol > 6.5 mmol/l and/or use of cholesterol-lowering drug(s)

### CCTA and FFR-CT

**Fig. 2:**
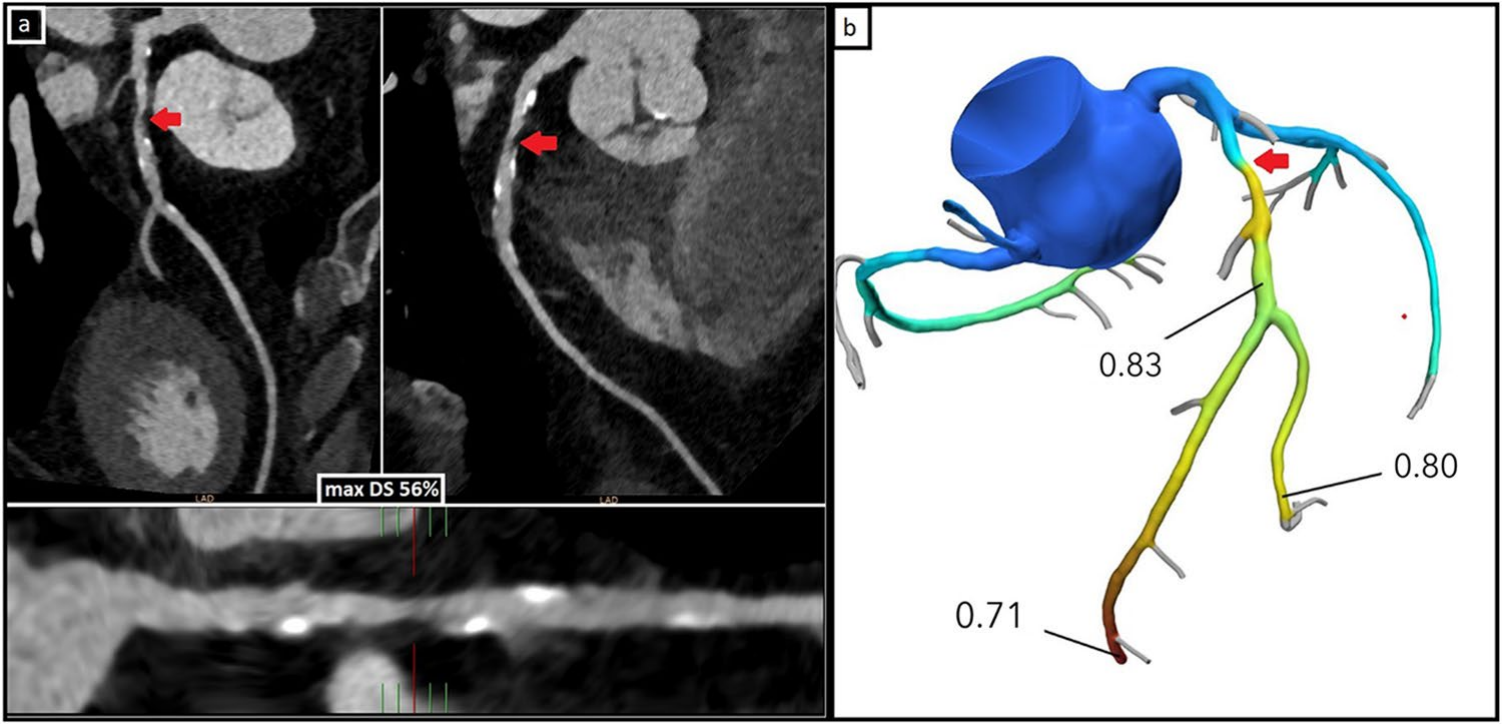
Example of significant stenosis on CCTA and negative FFR-CT result. **a** CCTA images of anatomically significant left anterior descending artery (LAD)-stenosis with maximal diameter reduction (max DS) 56%. **b** FFR-CT results for this CCTA. FFR-CT value after stenosis 0.83, which is negative for ischemia on FFR-CT. The red arrow points to the stenosis in all images. The patient was successfully treated with OMT only and was referred back to their general practitioner

Figure 2 shows an example of a lesion on CCTA with corresponding FFR-CT result. The number and severity of stenoses visualized on CCTA were comparable between the groups (Table 2). Sixty-two patients (45.6%) in the FFR-CT group had a positive FFR-CT for at least one coronary segment. In 8 patients, one or more coronary segments could not be analyzed with FFR-CT. These segments were eliminated from our analysis. None of these 8 patients underwent additional non-invasive imaging. Two CTs were rejected for FFR-CT, these patients were not included.

**Table 2:** Results of CCTA and FFR-CT analyses. Values are presented as *n* (%) or median (interquartile range). *CCTA* coronary computed tomography angiography, *FFR-CT* CT-derived fractional flow reserve, *RCA* right coronary artery, *RDP* Ramus Descendens posterior, *LAD* left anterior descending, *LCx* ramux circumflex, *RPL* right posterolateral artery, *PLCx* proximal left circumflex artery, *IM* Intermediate artery

	CCTA	FFR**-**CT	*p*-value
Calcium score			
Agatston score	118.5 (28.0–270.3)	121.5 (44.3–407.0)	0.130
Mass	23.0 (6.0–51.0)	22.5 (8.0–70.0)	0.366
Volume	102.0 (31.5–235.0)	116 (44.0–335.8)	0.095
Anatomical significance CCTA^a^			
RCA			
Proximal RCA (%)	35 (15.6)	18 (13.2)	0.535
Mid RCA (%)	56 (25.0)	21 (15.4)	0.032
Distal RCA (%)	22 (9.8)	14 (10.3)	0.885
RDP (%)	5 (2.2)	2 (1.5)	
Left main	8 (3.6)	4 (2.9)	
LAD			
Proximal LAD (%)	87 (38.8)	53 (39.0)	0.980
Mid LAD (%)	97 (43.3)	67 (49.3)	0.271
Distal LAD (%)	38 (17.0)	19 (14.0)	0.451
Diagonal 1 (%)	32 (14.3)	21 (15.4)	0.764
Diagonal 2 (%)	12 (5.4)	7 (5.1)	0.931
RCx			
Proximal Cx (%)	45 (20.1)	22 (16.2)	0.355
Mid Cx (%)	7 (3.1)	12 (8.8)	0.019
Marginal obtuse 1 (%)	21 (9.4)	8 (5.9)	0.238
Marginal obtuse 2 (%)	0 (0)	1 (0.7)	
Marginal obtuse 3 (%)	1 (0.4)	0 (0)	
RPL/PLCx (%)	9 (4.0)	5 (3.7)	0.871
IM (%)	17 (7.6)	11 (8.1)	0.864
Functional significance FFR**-**CT^b^			
FFR-CT RCA distal	–	0.84 (0.17)	
FFR-CT LAD distal	–	0.80 (0.11)	
FFR-CT LCx distal	–	0.82 (0.17)	
RCA			
Proximal RCA (%)	–	1 (0.7)	
Mid RCA (%)	–	2 (1.5)	
Distal RCA (%)	–	6 (4.4)	
RDP (%)	–	2 (1.5)	
Left main	–	2 (1.5)	
LAD			
Proximal LAD (%)	–	11 (8.1)	
Mid LAD (%)	–	16 (11.8)	
Distal LAD (%)	–	15 (11.0)	
Diagonal 1 (%)	–	15 (11.0)	
Diagonal 2 (%)	–	16 (11.8)	
LCx			
Proximal Cx (%)	–	5 (3.7)	
Mid Cx (%)	–	7 (5.1)	
Marginal obtuse 1 (%)	–	13 (9.6)	
Marginal obtuse 2 (%)	–	3 (2.2)	
Marginal obtuse 3 (%)	–	1 (0.7)	
RPL/PLCx (%)	–	6 (4.4)	
IM (%)	–	4 (2.9)	

^a^Anatomical significance based on CCTA is defined as CAD-RADS 3–5

^b^Functional significance based on FFR-CT is defined as FFR-CT ≤ 0.80

### Clinical management after CCTA and FFR-CT

**Fig. 3:**
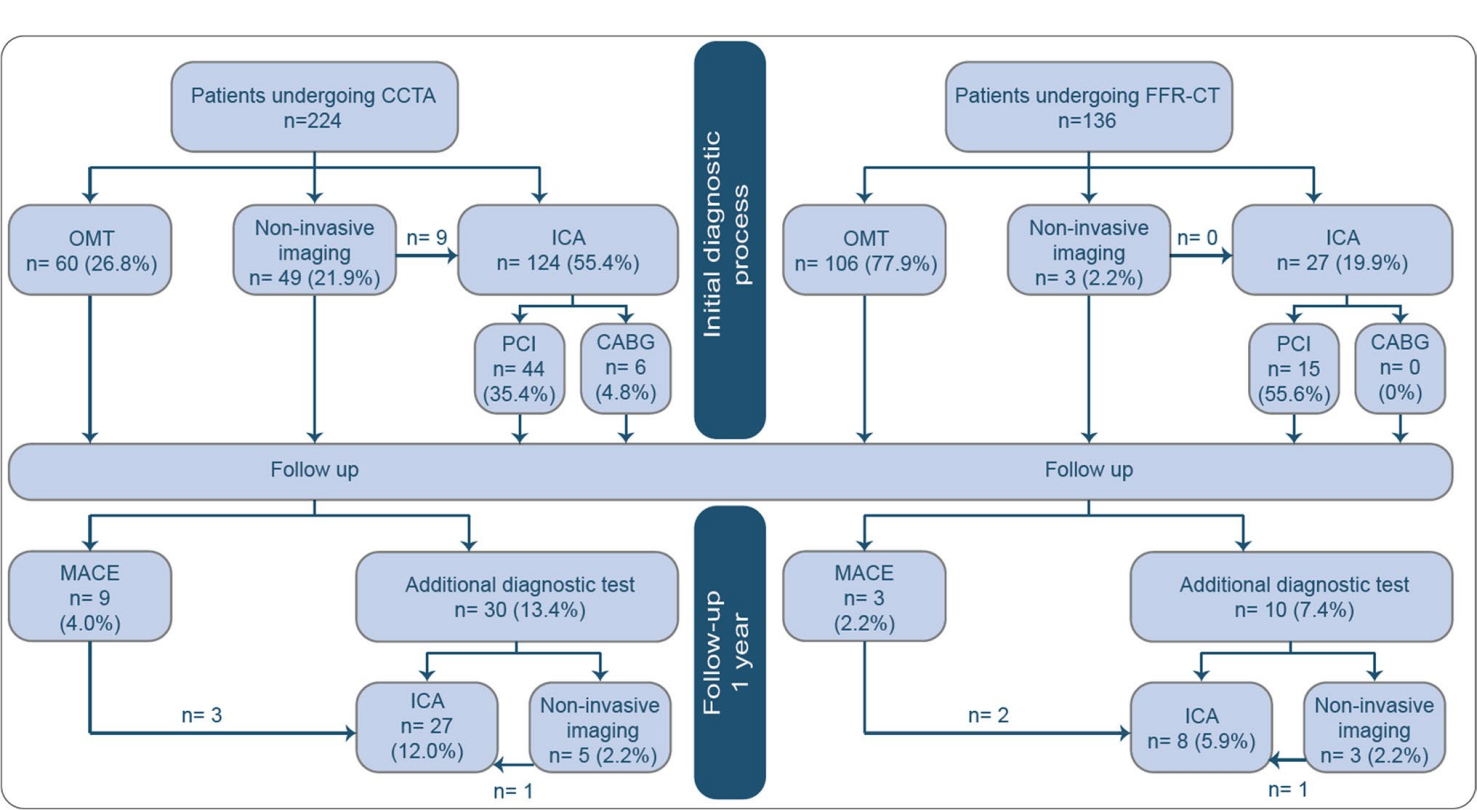
Clinical management by diagnostic strategy. Values are presented as *n* (%). CABG, coronary artery bypass grafting; CCTA, coronary computed tomography angiography; FFR-CT, computed tomography–derived fractional flow reserve; ICA, invasive coronary angiography; MACEs, major cardiovascular events; OMT, optimal medical treatment; PCI, percutaneous coronary intervention

Clinical management after CCTA with FFR-CT differed significantly from clinical management after CCTA alone (*p* < 0.001 for the three management options). In the CCTA group, 60 patients (26.8%) received optimal medical therapy (OMT) only, 49 (21.9%) underwent additional single positron emission CT (SPECT), and 115 (51.3%) underwent ICA as direct result of their CCTA. In the FFR-CT group, 106 patients (77.9%) received OMT only, three (2.2%) underwent SPECT, and 27 (19.9%) underwent ICA as direct result of their FFR-CT (Fig. 3). FFR-CT was positive in the majority (85.2%) of patients that underwent ICA.

In the CCTA group, SPECT was positive for cardiac ischemia in 21 patients (42.9%), of which 9 underwent ICA. Reported reasons to not perform ICA despite positive SPECT included small or distal areas of ischemia and resolution of symptoms. In the FFR-CT group, SPECT was negative in all three patients. Two of these had borderline positive FFR-CT values, the third an FFR-CT positive aberrant right coronary artery.

During diagnostic work-up, 124 patients (55.4%) in the CCTA group and 27 patients (19.9%) in the FFR-CT group underwent ICA (Fig. 3). Revascularization rates were 40.3% in the CCTA group and 55.6% in the FFR-CT group (*p* = 0.15). During ICA, invasive pressure measurements were obtained in 51 patients (41.1%) in the CCTA group and 16 (59.3%) in the FFR-CT group (*p* = 0.072). Revascularization was based on invasive pressure measurements in 38% of the CCTA group compared to 70% in the FFR-CT group (*p* = 0.05). One revascularized FFR-CT patient had a negative FFR-CT.

### Follow-up

**Fig. 4:**
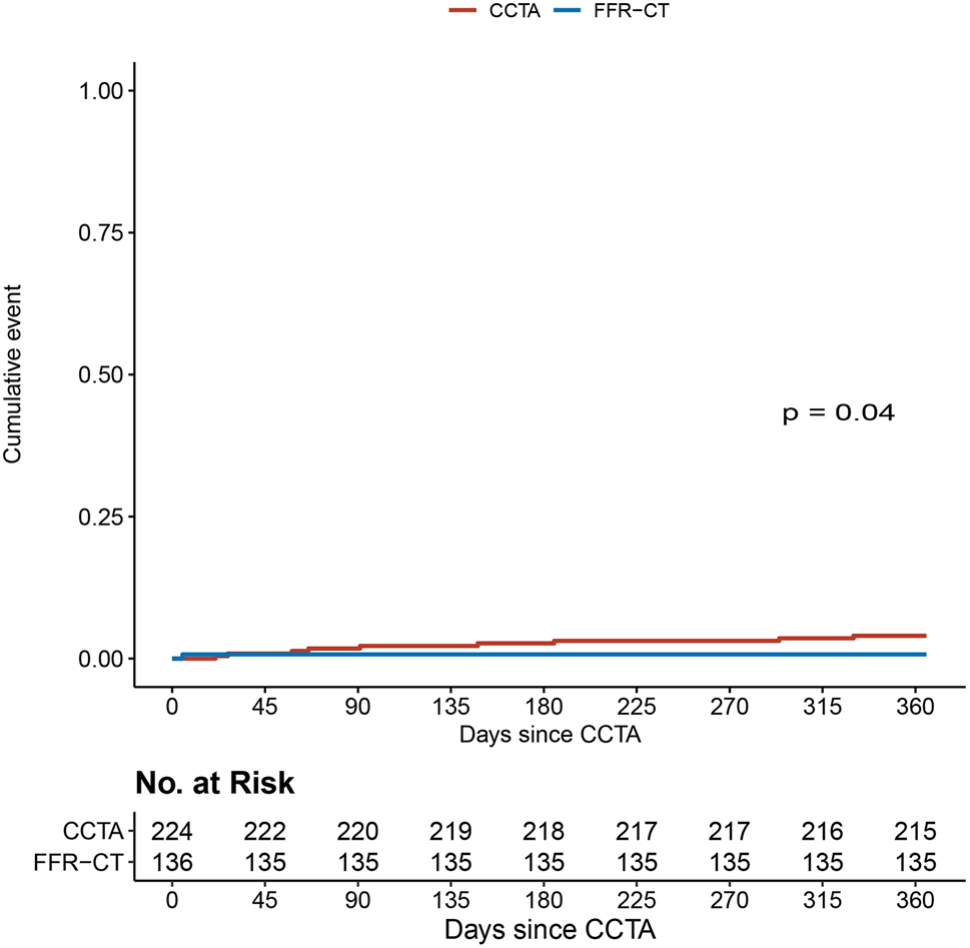
Time-to-event curve for major cardiovascular events. Shown is the time-to-event Kaplan–Meier curve of major cardiovascular events (MACEs). CCTA, coronary computed tomography angiography; FFR-CT, computed tomography derived FFR

During 1 year follow-up, 13 MACEs occurred in 12 patients, 9 in the CCTA group, and 3 in the FFR-CT group (Table 3). One patient in the CCTA group had two MACEs, aborted sudden cardiac death (due to hemorrhagic shock after splenic rupture) first, and death several hours later. The FFR-CTs of the patient with MI and one patient with urgent revascularization in the FFR-CT group were positive for ischemia, but ICA had not yet been performed. Kaplan–Meier survival curves for MACE (Fig. 4) and its individual components (Electronic Supplementary Materials 1-4 ) were constructed. Due to the low event rate, these did not provide additional insights.

**Table 3:** One-year clinical outcomes by diagnostic strategy. Values are presented as *n* = number of patients (%). *CCTA* coronary computed tomography angiography, *CVA* cardiovascular accident, *FFR-CT* computed tomography derived fractional flow reserve, *MACE* major adverse cardiovascular events—composite of all-cause mortality, aborted sudden cardiac death, myocardial infarction, and unplanned hospitalization for chest pain leading to urgent revascularization

	CCTA (*n* = 224)	FFR-CT (*n* = 136)	*p*-value
MACE (%)	9 (4.0)^a^	3 (2.2)	0.04
Death (%)	3 (1.3)	0 (0)	0.09
Cardiovascular death (%)	0 (0)	0 (0)	NA
Aborted sudden cardiac death (%)	1 (0.4)	0 (0)	0.33
Myocardial infarction^b^ (%)	6 (2.7)	1 (0.7)	0.17
Unplanned (urgent) revascularization	0 (0)	2 (1.5)	NA
CVA^c^ (%)	2 (0.9)	2 (1.5)	0.62

^a^One patient experienced two MACE events (aborted sudden cardiac death, died several hours later)

^b^Includes ST-elevated and non-ST-elevated myocardial infarction

^c^Includes ischemic stroke, hemorrhagic stroke, and transient ischemic attack

During follow-up, 27 patients in the CCTA group (12.0%) and 8 patients in the FFR-CT group (5.9%) underwent ICA. Revascularization rates during follow-up were 22% in the CCTA group and 50% in the FFR-CT group (*p* = 0.13). Most patients had not undergone ICA before (CCTA group 78%, FFR-CT group 75%). The number of FFR-CTs needed to avoid ICA or any additional diagnostic testing for one patient during both diagnostic work-up and follow-up was 2.5 and 1.8 respectively.

During the studied period, 24.6% (55/224 patients) of the CCTA group was revascularized, which was a significantly larger proportion than that of the FFR-CT group (18/136, 13.2%, *p* = 0.01). We did not observe differences regarding residual symptoms between both groups. Most patients were referred back to their general practitioner and did not return to the outpatient clinic.

## Discussion

In this study, the historical cohort for comparison of clinical management after CCTA alone allowed us to evaluate the impact of additional FFR-CT in a real-world setting. Our results show that FFR-CT analysis leads to patients receiving significantly more OMT alone, less additional invasive and non-invasive diagnostic testing, and less revascularization procedures. There was no significant difference in MACE between both groups. The reduction in additional (invasive) diagnostic testing combined with equally low MACE rates and similar rates of ICA and revascularization during follow-up shows that FFR-CT is a safe and effective gatekeeper for ICA.

The observed reduction in additional diagnostic testing with FFR-CT in routine clinical practice is supported by previous studies. In the PLATFORM-study, only 40% of the patients in whom invasive diagnostic strategy was planned underwent ICA after FFR-CT [19]. In the FORECAST-trial, the reduction in ICA procedures was 22% and the reduction of ICAs negative for obstructive CAD was 52% with a similar number of revascularization procedures in both groups [11]. Additionally, studies as the RIPCORD FFRCT and the ADVANCE registry showed that the functional information provided by FFR-CT increases the confidence in clinical decisions and reduces the need for additional diagnostic tests [7, 8].

The reduction in diagnostic procedures has several important benefits for patients and healthcare facilities. The main advantage for patients is the lower diagnostic burden, since FFR-CT can be derived from routine CCTA. It might spare additional diagnostic tests with the accompanying stress, travel and time cost, an additional consultation for the results, and the insecurity regarding the personal health in the meantime. CCTA with FFR-CT as gatekeeper to ICA is expected to result in less ICA procedures, and thus lower radiation and contrast exposure for a larger group of patients compared to CCTA alone, and less patients exposed to the previously mentioned risks of ICA. For healthcare facilities, less diagnostic tests reduces the pressure on planning capacity and personnel.

Beyond these benefits, implementing FFR-CT might result in a cost-reduction. The PLATFORM-study showed that FFR-CT, as gatekeeper towards an invasive diagnostic strategy, could lead to a cost-reduction of 20% or $2115 (*p* < 0.0001) within 90 days follow-up. However, using FFR-CT in patients with an indication for a noninvasive strategy led to significantly higher costs for FFR-CT if a cost weight of only a half CCTA was assumed for FFR-CT (cost increase 30% or $629, *p*0.02) [20]. The FORECAST trial described a non-significant cost difference between an FFR-CT-based strategy compared to standard care after 9 months of follow-up. Mean costs were slightly higher in the FFR-CT group (difference 8% or £114) whereas median costs were slightly higher in the standard care group (difference 12% or £70) [11].

In addition to diagnostic costs, less patients at risk for the complications of ICA will lead to a reduction in complications overall and healthcare costs associated with these complications. However, even if FFR-CT does not lead to a significant reduction in healthcare costs, the previously mentioned advantages of lower burden for patients and less pressure on hospital resources reflected in the results of this study support implementation of FFR-CT in routine healthcare.

It is noteworthy that only 37% of the patients with a positive FFR-CT in our cohort underwent ICA. In multiple other studies large proportions, around 40–50%, of patients with a positive FFR-CT do not undergo ICA as well [7, 21, 22]. In the ADVANCE registry, the on-site team referred less patients for ICA than the core lab would have, suggesting that clinical characteristics influence the interpretation of FFR-CT results [7]. In our study, many patients had a low pre-test risk of CAD. It is likely that this contributed to the high rate of conservative treatment strategies. Patients with positive FFR-CT that did undergo ICA might have had more severe CAD, but our study lacks power to determine whether lower mean FFR-CT values and more affected proximal coronary segments were present in this subgroup. Another noteworthy finding is that only 55.3% of the patients in the CCTA group underwent ICA during their diagnostic work-up. Approximately a quarter of these patients received no further diagnostic testing and only 43% of those with non-invasive testing results suggesting ischemia in this group underwent ICA. While it could be argued that this is not in accordance with the guidelines, additional (invasive) testing is not useful if revascularization is not expected [1]. Distal or difficult-to-approach CAD, small areas of ischemia, and resolution of symptoms were reasons to defer other (invasive) diagnostic tests and invasive treatment.

Despite the lower proportion of revascularization, we expect that the patients in the FFR-CT group were adequately treated. FFR measurements were obtained in less than half of the revascularized CCTA patients. Revascularization of intermediary lesions based on anatomical stenosis severity alone is associated with overtreatment and worse clinical outcomes [23, 24]. As mentioned before, current guidelines recommend functional assessment of anatomically significant stenoses, as it is known that the correlation between the anatomic and hemodynamic significance of CAD is not straightforward [1]. It is therefore likely that some revascularized patients in the CCTA group would not have been eligible for revascularization, had invasive measurements been performed. Trials as ORBITA, COURAGE, and ISCHEMIA have shown that the benefits of revascularization might be lower than previously thought [25–27]. Therefore, an initial conservative treatment strategy for negative FFR-CT results is expected to be safe even in the event of false negatives, as long as additional (invasive) assessment is reconsidered when symptoms persist or increase.

### Future perspectives

This study has shown that FFR-CT is a safe gatekeeper of ICA in assessing the functional severity of intermediate CAD. Future studies should focus on whether FFR-CT can substitute invasive diagnostic methods to assess the indication and approach of invasive treatment. Additionally, while we have shown that FFR-CT leads to an overall reduction in ICAs and invasive treatments, it is yet unclear whether an FFR-CT-based strategy is cost-effective compared to our current standard of care. Other less-invasive alternatives for FFR measurements have been developed, such as ICA-based FFR calculation (QFR, Medis medical imaging systems BV). FFR-CT and QFR have both been compared to the current standard, but not to each other. Moreover, the impact of these strategies on patient satisfaction and quality of life has not been assessed. The iCORONARY trial (clinicaltrials.gov: NCT04939207), for which inclusion started in March 2022, aims to assess all these topics.

### Strengths and limitations

The strength of the study is that it uses real-world data to assess the impact of availability of FFR-CT, which until now has mostly been used in clinical trials. The historical cohort ensures that the availability of FFR-CT is the main difference between both groups. Patient groups were comparable regarding baseline demographics, cardiac risk factors, and CAD severity on CCTA.

The study has some limitations inherent to the retrospective design. The decision to perform additional FFR-CT was not standardized. We expect that not all patients with anatomically significant stenoses on CCTA received FFR-CT while available, especially during the first months when FFR-CT was still unfamiliar. As these patients were not eligible for this study, we did not collect data regarding them or the reasoning behind this decision. However, as baseline and CT characteristics did not differ significantly between our groups, we do not expect this to have changed our outcomes. Demographics and clinical presentation were obtained from physicians’ reports only. These reports are not standardized, which led to vague or missing data and discrepancies in some records. However, there was no missing data regarding CCTA results, FFR-CT results, and clinical management. The single-center design might have reduced generalizability of the results. Finally, the groups were relatively small and overall MACE rate was low in both groups. We did assess whether follow-up until 31–12-2021 for all patients regardless of CCTA date would lead to a different conclusion regarding our main safety outcome. This was not the case.

## Conclusion

Addition of FFR-CT to CCTA leads to less additional diagnostic testing for hemodynamically significant CAD, both invasive and non-invasive, without an increase in MACE, additional diagnostic testing, or revascularization during a 1-year follow-up.

## Supplementary Information

Below is the link to the electronic supplementary material.

Supplementary file1 (PDF 132 KB)

## Abbreviations

CABG: Coronary artery bypass grafting
CAD: Coronary artery disease
CCTA: Coronary computed tomography angiography
FFR: Fractional flow reserve
FFR-CT: Computed tomography–derived FFR
ICA: Invasive coronary angiography
MACEs: Major adverse cardiac events
MI: Myocardial infarction
OMT: Optimal medical therapy
PCI: Percutaneous coronary intervention
QFR: Quantitative flow ratio
SCCT: Society of Cardiovascular Computed Tomography
SPECT: Single positron emission computed tomography

## Declarations

### Guarantor

The scientific guarantor of this publication is Martin J. Swaans.

### Statistics and biometry

One of the authors, Joyce Peper, has significant statistical expertise.

### Methodology

• retrospective

• observational

• performed at one institution

## References

[1] Neumann FJ, Sechtem U, Banning AP (2020). 2019 ESC Guidelines for the diagnosis and management of chronic coronary syndromes. Eur Heart J.

[2] Budoff MJ, Dowe D, Jollis JG (2008). Diagnostic performance of 64-multidetector row coronary computed tomographic angiography for evaluation of coronary artery stenosis in individuals without known coronary artery disease. Results from the prospective multicenter accuracy (assessment by coronary computed tomographic angiography of individuals undergoing invasive coronary angiography) trial. J Am Coll Cardiol.

[3] Pijls NHJ, Fearon WF, Tonino PAL (2010). Fractional flow reserve versus angiography for guiding percutaneous coronary intervention in patients with multivessel coronary artery disease: 2-Year follow-up of the FAME (fractional flow reserve versus angiography for multivessel evaluation) study. J Am Coll Cardiol.

[4] Bech GJW, De Bruyne B, Pijls NHJ (2001). Fractional flow reserve to determine the appropriateness of angioplasty in moderate coronary stenosis: a randomized trial. Circulation.

[5] Peper J, Schaap J, Kelder JC (2021). Added value of computed tomography fractional flow reserve in the diagnosis of coronary artery disease. Sci Rep.

[6] Tavakol M, Ashraf S, Brener SJ (2012). Risks and complications of coronary angiography: a comprehensive review. Glob J Health Sci.

[7] Fairbairn TA, Nieman K, Akasaka T (2018). Real-world clinical utility and impact on clinical decision-making of coronary computed tomography angiography-derived fractional flow reserve: lessons from the ADVANCE Registry. Eur Heart J.

[8] Curzen NP, Nolan J, Zaman AG, Nørgaard BL, Rajani R (2016). Does the routine availability of CT–derived FFR influence management of patients with stable chest pain compared to ct angiography alone?: The FFRCT RIPCORD Study. JACC Cardiovasc Imaging.

[9] Lu MT, Ferencik M, Roberts RS (2017). Noninvasive FFR derived from coronary CT angiography: management and outcomes in the PROMISE trial. JACC Cardiovasc Imaging.

[10] Driessen RS, Danad I, Stuijfzand WJ (2019). Comparison of coronary computed tomography angiography, fractional flow reserve, and perfusion imaging for ischemia diagnosis. J Am Coll Cardiol.

[11] Curzen N, Nicholas Z, Stuart B (2021). Fractional flow reserve derived from computed tomography coronary angiography in the assessment and management of stable chest pain: The FORECAST randomized trial. Eur Heart J.

[12] Nakanishi R, Budoff MJ (2016). Noninvasive FFR derived from coronary CT angiography in the management of coronary artery disease: technology and clinical update. Vasc Health Risk Manag.

[13] Gaur S, Achenbach S, Leipsic J (2013). Rationale and design of the HeartFlowNXT (HeartFlow analysis of coronary blood flow using CT angiography: NeXt sTeps) study. J Cardiovasc Comput Tomogr.

[14] De Bruyne B, Fearon WF, Pijls NHJ (2014). Fractional flow reserve–guided PCI for stable coronary artery disease. N Engl J Med.

[15] Brandt V, Schoepf UJ, Aquino GJ (2022). Impact of machine-learning-based coronary computed tomography angiography–derived fractional flow reserve on decision-making in patients with severe aortic stenosis undergoing transcatheter aortic valve replacement. Eur Radiol.

[16] Leipsic J, Abbara S, Achenbach S (2014). SCCT guidelines for the interpretation and reporting of coronary CT angiography: a report of the Society of Cardiovascular Computed Tomography Guidelines Committee. J Cardiovasc Comput Tomogr.

[17] Abbara S, Blanke P, Maroules CD (2016). SCCT guidelines for the performance and acquisition of coronary computed tomographic angiography: a report of the society of Cardiovascular Computed Tomography Guidelines Committee: Endorsed by the North American Society for Cardiovascular Imaging (NASCI). J Cardiovasc Comput Tomogr.

[18] Austen WG, Edwards JE, Frye RL (1975). AHA COMMITTEE REPORT: a reporting system on patients evaluated for coronary artery disease report of the ad hoc committee for grading of coronary artery. Circulation.

[19] Pontone G, Guaricci AI, Palmer SC (2020). Diagnostic performance of non-invasive imaging for stable coronary artery disease: a meta-analysis. Int J Cardiol.

[20] Hlatky MA, De Bruyne B, Pontone G (2015). Quality-of-life and economic outcomes of assessing fractional flow reserve with computed tomography angiography: PLATFORM. J Am Coll Cardiol.

[21] Nørgaard BL, Terkelsen CJ, Mathiassen ON (2018). Coronary CT angiographic and flow reserve-guided management of patients with stable ischemic heart disease. J Am Coll Cardiol.

[22] Kvist TV, Nørgaard BL, Bøtker HE (2021). Computed tomography-derived fractional flow reserve in patients with chronic coronary syndrome: a real-world cohort study. J Comput Assist Tomogr.

[23] Zimmermann FM, Ferrara A, Johnson NP (2015). Deferral vs. performance of percutaneous coronary intervention of functionally non-significant coronary stenosis 15-year follow-up of the DEFER trial. Eur Heart J..

[24] Tonino PAL, De Bruyne B, Pijls NHJ, et al. 2009 Fractional flow reserve versus angiography for guiding percutaneous coronary intervention. N Engl J Med. 360(3)10.1056/NEJMoa0807611 10.1056/NEJMoa0807611 19144937

[25] Al-Lamee R, Thompson D, Dehbi HM, et al. 2018 Percutaneous coronary intervention in stable angina (ORBITA): a double-blind, randomised controlled trial. Lancet. 391(10115). 10.1016/S0140-6736(17)32714-9 10.1016/S0140-6736(17)32714-9 29103656

[26] Maron DJ, Hochman JS, Reynolds HR (2020). Initial invasive or conservative strategy for stable coronary disease. N Engl J Med.

[27] Boden WE, O’Rourke RA, Teo KK, et al. 2007 Optimal medical therapy with or without PCI for stable coronary disease. N Engl J Med. 356(15). 10.1056/NEJMoa070829 10.1056/NEJMoa070829 17387127

