# Supplementary material for: Real world impact of added FFR-CT to coronary CT angiography on clinical decision-making and patient prognosis – IMPACT FFR study
Source: Eur Radiol. 2023 Mar 15;33(8):5465–75. doi: 10.1007/s00330-023-09517-z (PMC10326083; doi:10.1007/s00330-023-09517-z)
Supplement: Supplementary file 1 — Supplementary file1 (PDF 132 KB) [file 330_2023_9517_MOESM1_ESM.pdf]

Supplementary material

Supplementary figure 1: Time-to-event curve for all-cause mortality

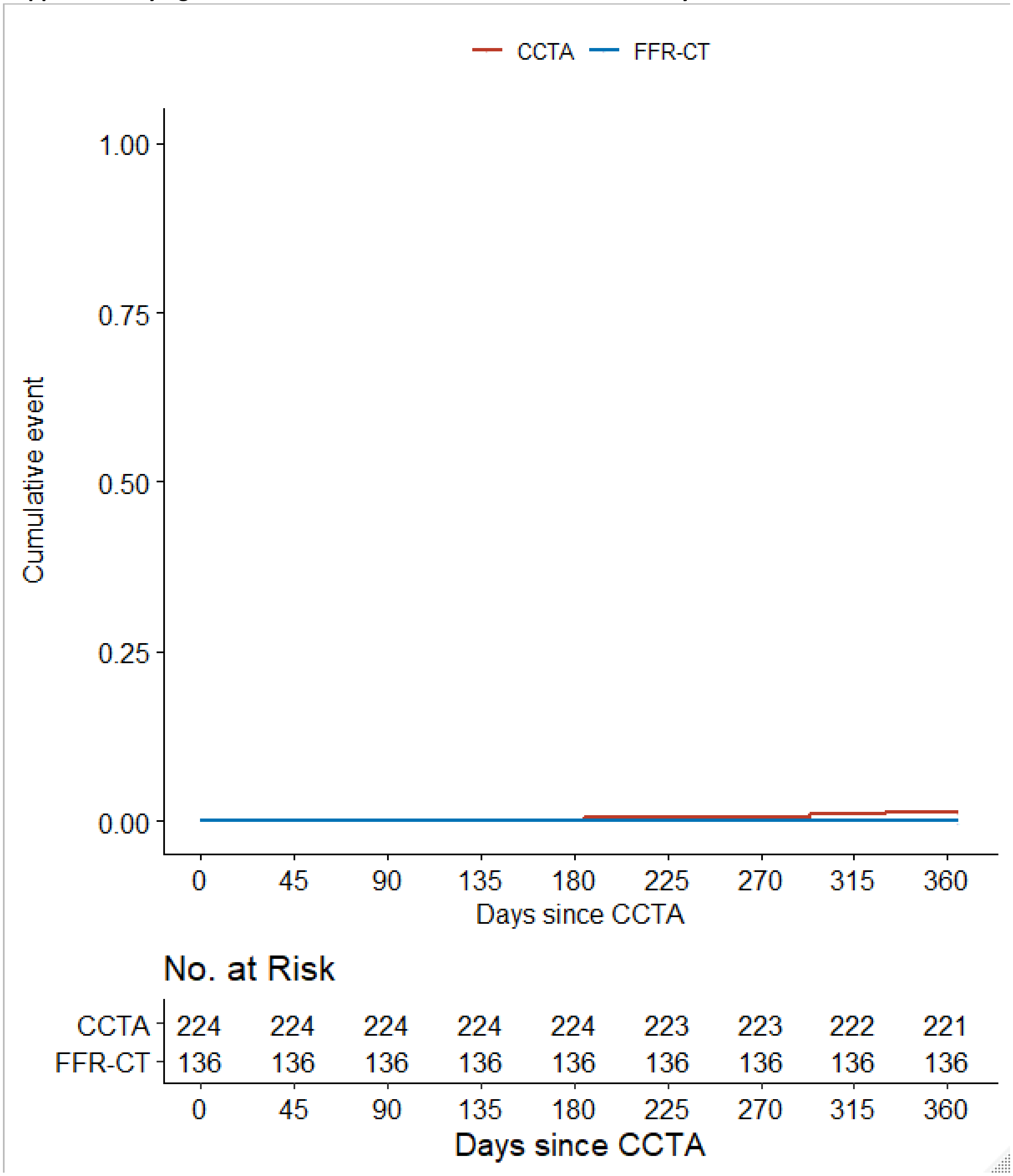

Shown is the time-to-event Kaplan-Meier curve of all-cause mortality. CCTA; coronary computed tomography angiography, FFR-CT; computed tomography derived fractional flow reserve.

Supplementary figure 2: Time-to-event curve for aborted sudden cardiac death

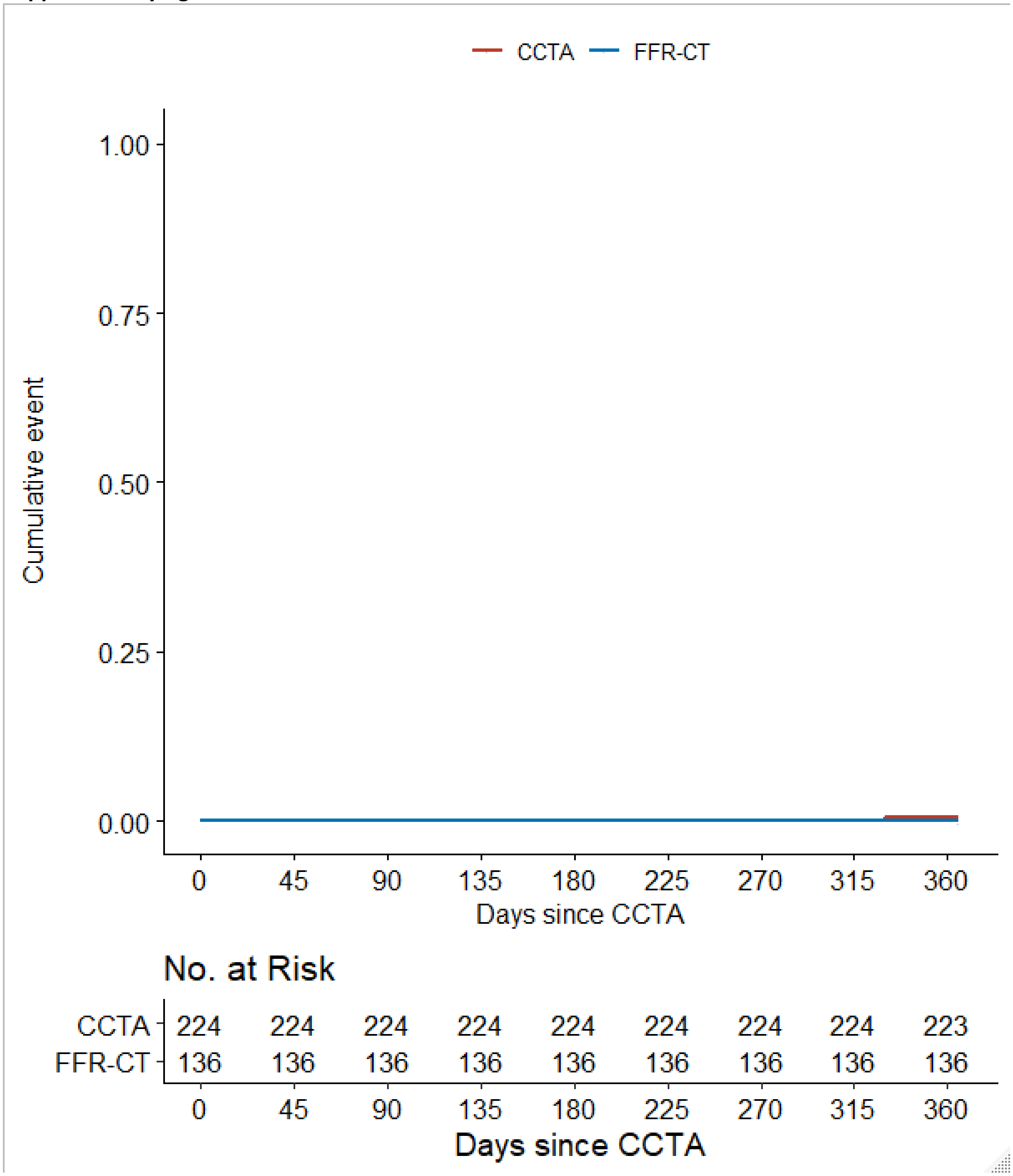

Shown is the time-to-event Kaplan-Meier curve of aborted sudden cardiac death. CCTA; coronary computed tomography angiography, FFR-CT; computed tomography derived fractional flow reserve.

Supplementary figure 3: Time-to-event curve for myocardial infarction

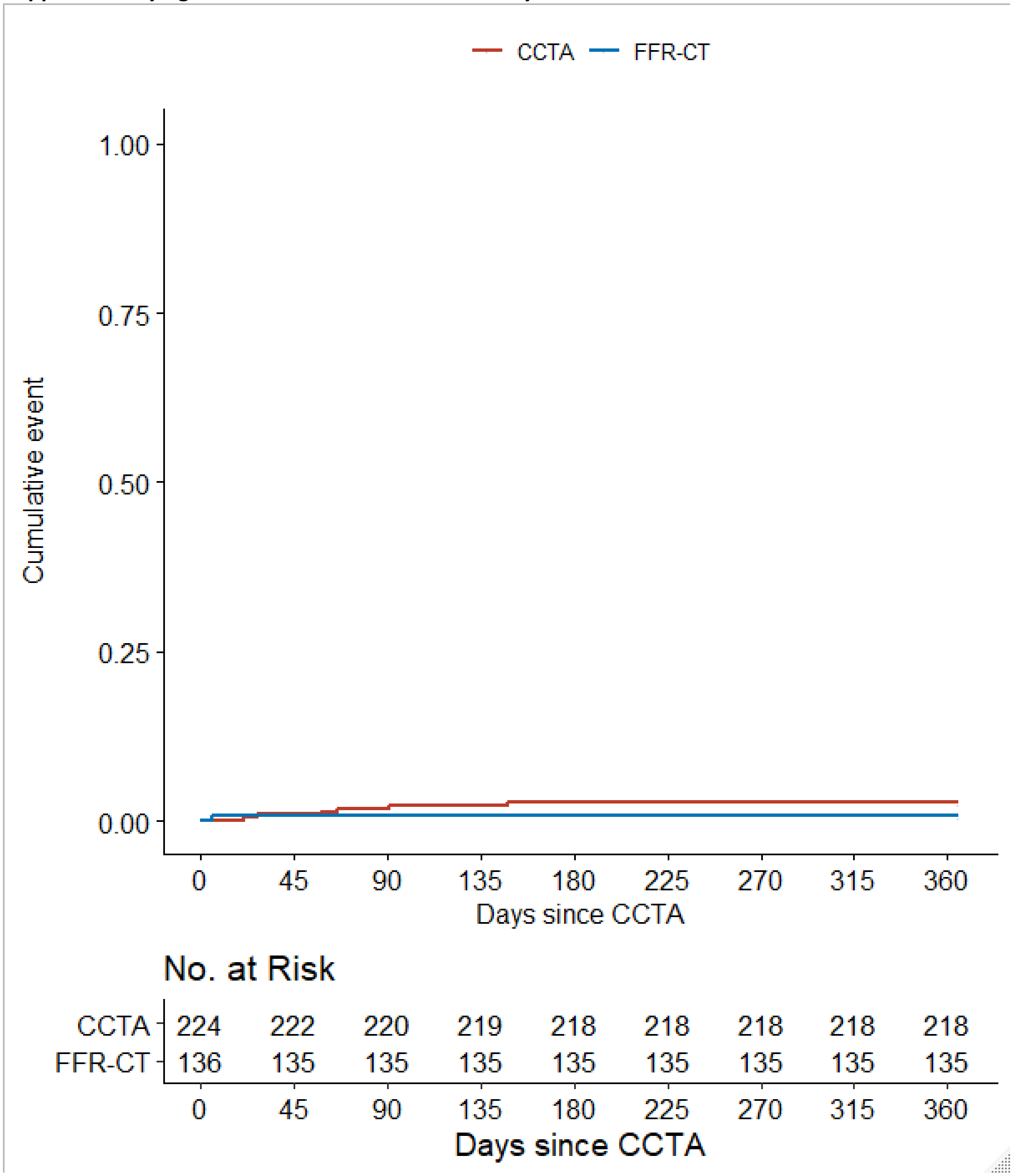

Shown is the time-to-event Kaplan-Meier curve of myocardial infarction. CCTA; coronary computed tomography angiography, FFR-CT; computed tomography derived fractional flow reserve.

Supplementary figure 4: Time-to-event curve for cerebrovascular accidents

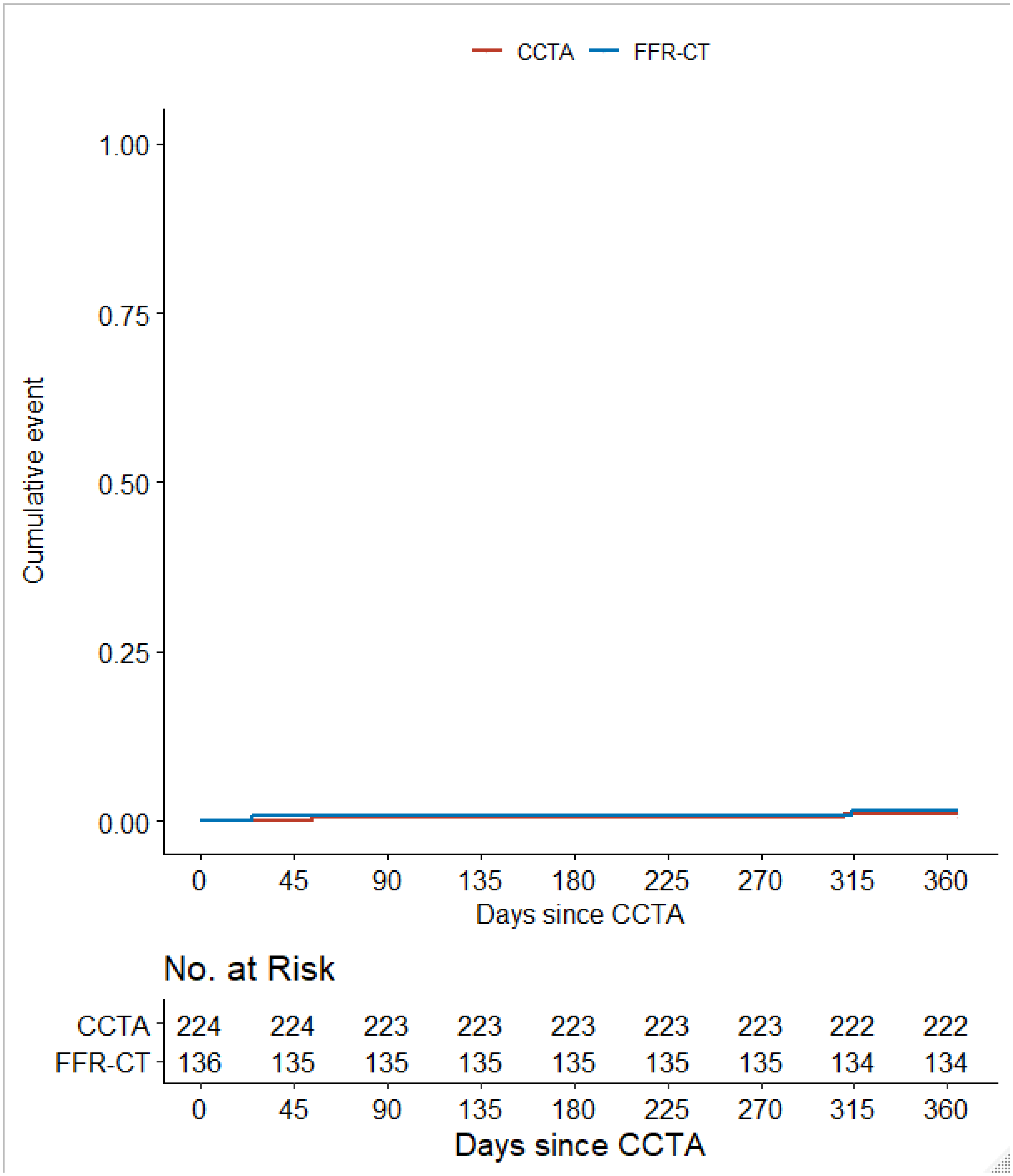

Shown is the time-to-event Kaplan-Meier curve of cerebrovascular accidents (CVA). CCTA; coronary computed tomography angiography, FFR-CT; computed tomography derived fractional flow reserve.
